# Parental Ability to Identify Severe Illnesses in Their Children

**DOI:** 10.1001/jamanetworkopen.2025.59998

**Published:** 2026-02-17

**Authors:** Hilla Pöyry, Jenni Turunen, Elisa Ritola, Sofia Hartikainen, Joni Palviainen, Ilona Liimatta, Ulla Koskela, Tytti Pokka, Marjo Renko, Niko Paalanne, Otto Helve, Mysore V. Tejesvi, Terhi Ruuska-Loewald

**Affiliations:** 1 Department of Pediatrics and Adolescent Medicine, Oulu University Hospital, Oulu, Finland; 2 Medical Research Center, Oulu University Hospital, Oulu, Finland; 3 Biocenter Oulu, University of Oulu, Oulu, Finland; 4 Department of Pediatrics, The University of Eastern Finland and Kuopio University Hospital, Finland; 5 Children’s Hospital, Pediatric Research Center, University of Helsinki and Helsinki University Hospital, Helsinki, Finland

## Abstract

**Importance:**

Early parental recognition of severe illness in children and adolescents is crucial for timely management and improved outcomes in pediatric emergency care.

**Objective:**

To assess how accurately parents can identify severe illness in their children using a questionnaire completed shortly after arrival at the emergency department (ED).

**Design, Setting, and Participants:**

This diagnostic study was conducted in a tertiary pediatric ED in northern Finland. Data were collected in 2019 to 2021, and this analysis was conducted in May 2024 to May 2025. Children and adolescents whose parents completed the questionnaire before physician assessment were included.

**Exposures:**

A structured, 36-item parental questionnaire assessing symptoms and the child or adolescent’s overall condition.

**Main Outcomes and Measures:**

Severe illness was defined as 1 or more of the following: admission to the pediatric intensive care unit, hospital treatment of more than 24 hours, need for intravenous or nasogastric fluids, need for intravenous antibiotics for more than 24 hours, oxygen saturation less than 93% or the need for inhaled medications, anaphylactic shock, intoxication requiring hospital admission, or surgical intervention. Sensitivity and specificity were calculated for each question. To identify parental triage questions with the strongest diagnostic value, a machine learning analysis was conducted.

**Results:**

Among 2375 included children and adolescents (mean [SD] age, 5.4 [4.6] years; 1140 female [48.0%]), 567 individuals (23.9%) met criteria for severe illness. Moderate to high parental worry showed the highest sensitivity (91.0% [95% CI, 88.3%-93.2%]) but the lowest specificity (17.5% [95% CI, 15.8%-19.4%]). Other specific pediatric questions demonstrated modest diagnostic accuracy with limited additional value. The machine learning model (area under the receiver operating characteristic curve, 0.71; 95% CI, 0.65-0.77) identified parental worry (feature importance score, 0.047), parent assessments of child or adolescent’s general condition (feature importance score, 0.046), and need for treatment (feature importance score, 0.141) as the strongest predictors of hospital admission.

**Conclusions and Relevance:**

In this study, parental worry identified most cases of severe illness but had low specificity. These findings suggest that while parental concern may serve as an initial screening indicator, it should be complemented by clinical evaluation and objective measures to avoid unnecessary escalation of care.

## Key Points

**Question:**

Can parents accurately identify severe illnesses in their children before emergency department evaluation using a symptom questionnaire?

**Findings:**

In this diagnostic study of 2375 children and adolescents, moderate to high parental worry showed high sensitivity but low specificity for identifying severe illness, defined as conditions requiring extended observation, specific treatment, or pediatric intensive care unit care. Additional symptom-based questions provided limited incremental diagnostic value.

**Meaning:**

These findings suggest that parental worry may serve as an efficient initial screening indicator for severe illness, but its low specificity underscores the need for complementary clinical assessment.

## Teaser

This diagnostic study assesses the ability of parents to identify severe illness in their children using a questionnaire at the emergency department.

## Introduction

Severe pediatric infections have become rare in high-income countries.^1^ Most children with acute illness experience a self-limiting disease that can be managed at home.^2^ Nonetheless, pediatric emergency department (ED) visits are increasing.^3^ Thus, early recognition of severe illness in children is challenging, even for health care professionals. Illness severity scores and triage guidelines have been developed to improve the identification of children with severe illness.^4,5,6^

Digital tools may improve the detection of severe illness in children.^7^ Although virtual care is becoming increasingly popular, there are limited data on how accurately parents can assess symptoms or identify severe illnesses in their children. A high level of parental concern and parent gut feeling that “this illness is different” have been associated with a child’s severe febrile illness in primary care settings.^8,9,10,11^ Collaboration between the child’s family and the treating team can improve the timely recognition of severe infection.^12^ Clinicians should actively listen to parents’ concerns and observations to improve outcomes for pediatric patients.^13^ There is an urgent need to improve the understanding of whether parental worry adds diagnostic value to recognizing severe illnesses.^14^

In this study, we evaluated the diagnostic accuracy of parental triage questions. We applied machine learning to identify which parental observations best predicted severe illness in children and adolescents with acute illness presenting to a pediatric ED.

## Methods

### Ethical Approval and Consent

The Oulu University Hospital Ethics Committee, Oulu, Finland, reviewed and approved the research plan for this diagnostic study. According to institutional policy, written informed consent was not required for observational diagnostic-accuracy studies. Clinical trial registration was not required. This study is reported following the Standards for Reporting of Diagnostic Accuracy (STARD) reporting guideline for diagnostic accuracy studies.

### Study Design and Oversight

**Figure 1.**
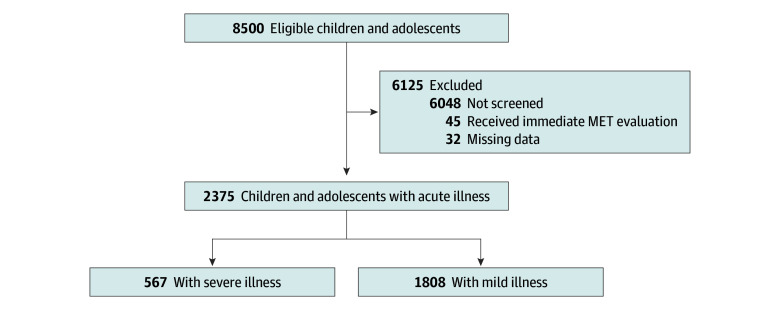
Study Flowchart The flow of children and adolescents with acute illness presenting to the pediatric emergency department during the study period is presented. Of 8500 children and adolescents, parents of 6048 patients were not offered the parental triage survey. A total of 2452 child or adolescent–parent pairs were recruited, among whom 45 patients required immediate medical team evaluation and 32 patients had missing identification codes or unanswered questionnaires. The final analytic sample consisted of 2375 children and adolescents, including 567 patients (23.9%) with severe illness and 1808 patients (76.1%) with mild illness. MET indicates medical emergency team.

The aim of this study was to investigate the diagnostic accuracy of different parental triage questions (Figure 1). The study cohort comprised 2375 child or adolescent–parent pairs who visited a pediatric ED at a university hospital in northern Finland between August 2019 and July 2021. The pediatric ED provides primary, secondary, and tertiary care services for children and adolescents aged 0 to 16 years with acute illness. During the study period, 8500 children and adolescents visited the ED. No systematic exclusion criteria were applied other than the inability to communicate in Finnish. The parental questionnaire was not offered to parents of 6048 children and adolescents due to peak crowding or limited staff availability. Parental refusal was uncommon. This analysis was conducted from May 8, 2024, to May 25, 2025. This was an investigator-initiated diagnostic cohort study.

### Parental Triage Questionnaire

Parents who agreed to participate were advised to complete the questionnaire alone or with a partner. The questionnaire included 36 items based on the child or adolescent’s symptoms and the parent’s observations (eAppendix 1 in Supplement 1). It included items derived from tools previously used by health care professionals to assess young children and adolescents (Acute Illness Observation Scale) (eAppendix 2 in Supplement 1). Other questions reflected clinically relevant symptoms and previously published illness severity scales.^5,15,16,17,18^ Parents were not provided any additional instructions and could complete the questionnaire only once during 1 visit.

### Definition of Severe Illness

Severe illness was defined as 1 or more of the following: admission to the pediatric intensive care unit (PICU), hospital treatment lasting more than 24 hours, need for intravenous or nasogastric fluids, need for intravenous antibiotics for more than 24 hours (in hospital or at home), oxygen saturation less than 93% or the need for inhaled medications, anaphylactic shock, intoxication requiring hospital or PICU admission, or surgical intervention. We reviewed the patient’s medical records to obtain medical histories and determine outpatient and hospital care, readmissions, and diagnoses.

### Sample Size and Diagnostic Test Accuracy

Before the study, we estimated the minimum required sample size to achieve the target sensitivity and specificity.^19^ We assumed that the most effective parental questions should be associated with an increase in sensitivity (total minimum sample size required: 257 children and adolescents) and specificity (total minimum sample size required: 2310 children and adolescents) for severe illnesses from 90% to 95%. Thus, we decided to recruit at least 2300 child or adolescent–parent pairs. We compared the diagnostic accuracy of parental questions in recognizing severe illness in children and adolescents with acute illness. We calculated the sensitivity and specificity of the questions, with 95% CIs, for all children and adolescents and for children younger than 2 years with febrile illness.

### Statistical Analysis

#### Application of Machine Learning on Questionnaire Data to Predict Patient Outcomes

We used machine learning on questionnaire data to predict patient outcomes. We separately analyzed 2 datasets: all patients and children younger than 2 years with febrile illness. We omitted patient characteristics and nonquestionnaire or irrelevant data from the analysis. Outcome variables were grouped into simple no and yes classes because some subclasses had small class sizes.

We used scikit-learn (version 1.3.0) in Python version 3.13.3 (Python Software Foundation) to conduct machine learning analysis.^20^ Pandas version 2.2.3 was used to manipulate data in Python formatted using Excel software version 2504 (Microsoft Corp), and NumPy version 2.2.5 was used to reorder important features. As preprocessing, we removed nonquestionnaire data, aside from specific outcomes to predict, from the data frame. Only children and adolescents with a fully completed questionnaire without any missing values were included in the analysis.

Data were categorical and originally encoded; therefore, no further preprocessing was performed. Datasets were randomly split into training and test sets, with 80% of patients in the training set and 20% of patients in the test set. Because most variables had uneven class sizes, the split was performed via stratification to ensure that the same proportion of classes was used during data splitting.

We trained 5 algorithms, including random forest, linear support vector classification, gradient boosting, *K*-nearest neighbors, and extra trees, to determine the best-performing classifier. Hyperparameter tuning was performed using randomized search cross-validation (eTable 1 in Supplement 1), and the best-performing parameters were refitted to the final classifier. We used 100 iterations during the tuning, nested cross-validation with 10-folds, and a random state of 123. The best-performing classifier was trained on the gradient-boosting algorithm (eTable 2 in Supplement 1). Given that the purpose of this study was to data mine the most important features associated with outcomes rather than create an effective classifier to estimate unknown data, the best-performing classifier was not externally validated on a new dataset.

We generated receiver operating characteristic (ROC) curves and bar plots of the ten most important features for classification. In this study, area under the ROC curve (AUROC) values of 0.5 to 0.6, >0.6 to 0.7, >0.7 to 0.8, >0.8 to 0.9, and >0.9 were considered to have virtually no accuracy, poor accuracy, moderately good accuracy, good accuracy, and excellent accuracy, respectively. All figures were drawn using Matplotlib version 3.8.4.^21^ In addition to using machine learning, we used forward stepwise multivariate logistic regression analysis of parental triage questions in predicting severe illness (eTable 3 in Supplement 1).

#### Exploratory Analysis of a Parental Score in Recognizing Severe Illness

To assess whether combining key items would be associated with improved discrimination beyond single-question performance, we then constructed a simple score from 3 parental questions with the highest feature importance in the machine learning analysis (Q1, Q5, and Q35). The discriminative ability of the summed score was evaluated using ROC curves and AUROC values with 95% CIs (eTables 3 and 4 in Supplement 1).

## Results

### Patient Characteristics

A total of 2375 child or adolescent–parent pairs completed the questionnaire before the physician assessment (child or adolescent mean [SD] age, 5.4 [4.6] years; 1140 female [48.0%]) (Figure 1; Table 1). Of 2452 families approached, 77 were excluded due to the need for immediate care (45 families) or an empty questionnaire (32 families). Of 2375 included patients, a total of 841 children and adolescents (35.4%) were younger than 2 years and 493 children and adolescents (20.8%) had an underlying illness, most commonly allergy or asthma. In 769 children and adolescents (32.4%), symptoms started within 24 hours of the family’s arrival at the ED. Most children and adolescents (1056 patients [44.5%]) were walk-in patients without a referral, while 643 patients (27.1%) came after a referral from a primary care physician, 617 patients (26.0%) after calling the phone triage service, and 56 patients (2.4%) by emergency ambulance. Overall, 537 children and adolescents (22.6%) were admitted to the hospital and 28 (1.2%) to the PICU. The most common diagnoses were upper respiratory tract infection (271 patients [11.4%]), abdominal pain (148 patients [6.2%]), fever of unknown origin (144 patients [6.1%]), and bronchiolitis or wheezing (125 patients [5.3%]).

**Table 1.** Baseline Patient Characteristics

Characteristic	Patients, No. (%) (N = 2375)
Demographics	
Age	
Mean (SD), y	5.4 (4.6)
<2 y	841 (35.4)
<2 y With fever	354 (14.9)
<1 mo	94 (4.0)
Sex	
Female	1140 (48.0)
Male	1235 (52.0)
Coexisting illness	
Allergy	178 (7.5)
Preterm birth	103 (4.3)
Asthma	76 (3.2)
Migraine	32 (1.3)
Heart disease	22 (0.9)
Epilepsy	19 (0.8)
Type 1 diabetes	14 (0.6)
Other	184 (7.7)
None	1882 (79)
Disease characteristics	
Symptom duration	
No. with data	1804
Mean (SD), h	76 (324)
<3 h	241 (13.4)
3-24 h	528 (29.3)
1-3 d	605 (33.5)
>3 d	430 (23.8)
Fever during illness	
No. with data	2332
Yes	929 (39.8)
Probably yes	121 (5.0)
No or probably no	1282 (54.9)
ED visit details	
First visit	2240 (94.3)
Revisit	116 (4.9)
Control visit	19 (0.8)
Referral source	
No. with data	2373
Walk-in	1056 (44.5)
Primary care	643 (27.1)
Triage phone	617 (26.0)
Ambulance	56 (2.4)
Hospital transfer	1 (<0.1)
Diagnoses at ED (*ICD-10*)	
Respiratory tract infections	625 (26.3)
Upper respiratory tract infection	271 (11.4)
Bronchiolitis or viral wheezing	125 (5.3)
Laryngitis	98 (4.1)
Pneumonia	24 (1.0)
Other common infections	431 (18.1)
Otitis media	103 (4.3)
Viral infection	89 (3.7)
Pyelonephritis	64 (2.7)
Enteritis	57 (2.4)
Symptom-based diagnoses	561 (23.6)
Abdominal pain	148 (6.2)
Fever NAS	144 (6.1)
Crying infant	77 (3.2)
Seizures	38 (1.6)
Headache	31 (1.3)
Dyspnea	28 (1.2)
Vomiting	23 (1.0)
Conditions treated surgically	43 (1.8)
Appendicitis	18 (0.8)
Testicular torsion	8 (0.3)
Type 1 diabetes	23 (1.0)
Hospital admissions and outcomes	
No. with data	2333
Not admitted	1810 (77.6.)
Admitted to ward	
<24 h	141 (6.0)
1-3 d	238 (10.2)
>3 d	116 (5.0)
PICU admission	28 (1.2)
Recontact within 4 wk	237 (10.2)

Abbreviations: ED, emergency department; *ICD-10*, *International Statistical Classification of Diseases and Related Health Problems, Tenth Revision*; NAS, no apparent source; PICU, pediatric intensive care unit.

### Children and Adolescents With Severe Illness

Altogether 567 children and adolescents (23.9%) met at least 1 criterion for severe illness. The following criteria for a severe illness were used: PICU admission (28 patients), hospital treatment for more than 24 hours (369 patients), need for intravenous or nasogastric fluids (148 patients), need for intravenous antibiotics for more than 24 hours (140 patients), respiratory distress with oxygen saturation less than 93% (45 patients), need for inhaled medications (190 patients), anaphylactic shock (9 patients), intoxication requiring admission (2 patients), and need for surgical treatment (43 patients).

### Response Rates for Parental Triage Questions

**Figure 2.**
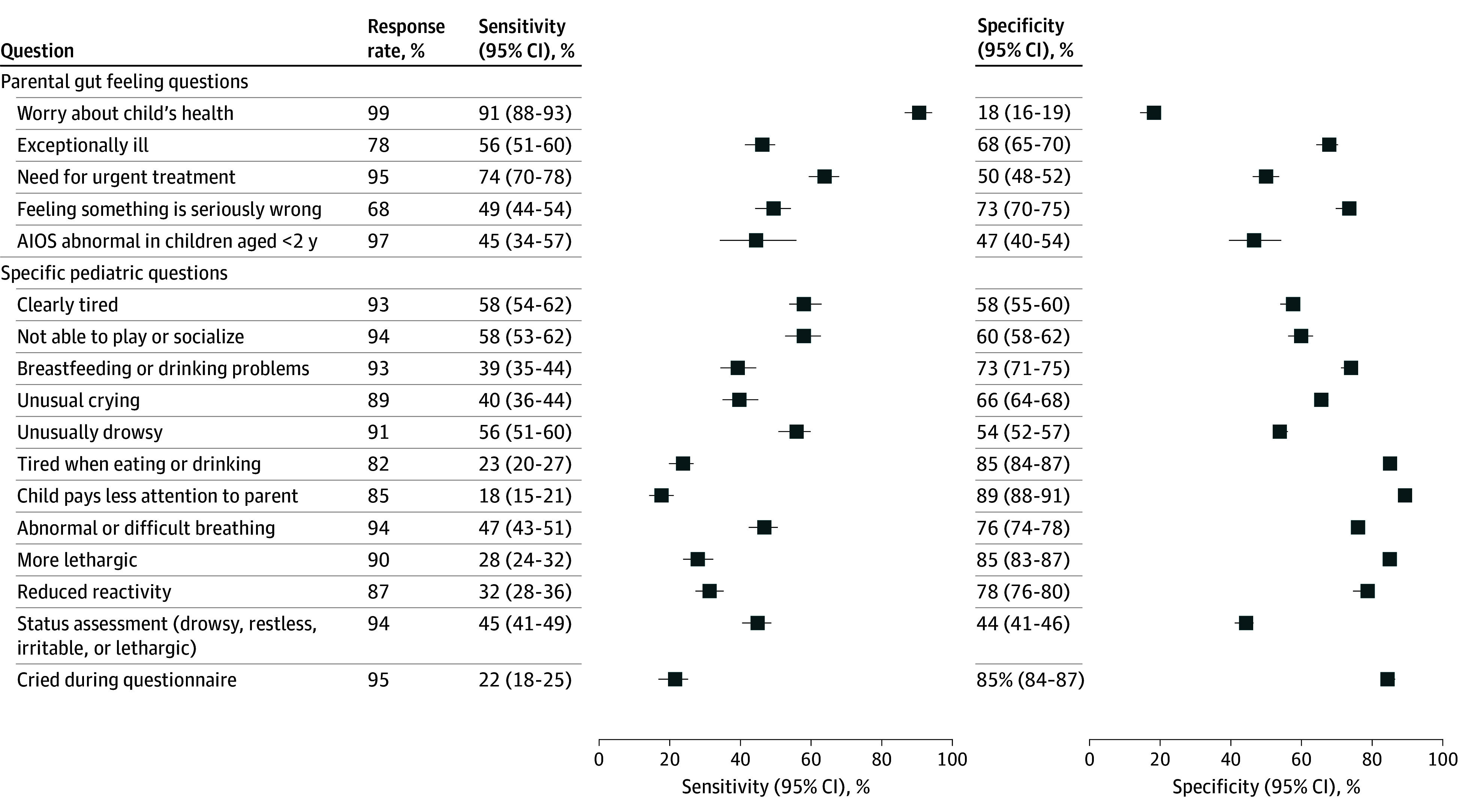
Forest Plot of Response Rates, Sensitivity, and Specificity of Parental Triage Questions for Identifying Severe Illness Sensitivity and specificity are shown with 95% CIs. *Yes* responses to questions were considered a positive test result. AIOS indicates Acute Illness Observation Scale.

Most parents aimed to complete the whole questionnaire, with 2265 parents (95.4%) responding to the final question (Figure 2 and Table 2). The question with the highest response rate was regarding the degree of parental worry (Q1; 2358 parents [99.3%]) (Figure 2 and Table 2). Most parents gave their opinions about the need for treatment for their child (Q5; 2267 parents [95.5%]) and whether they considered their child well enough to play and socialize as usual (Q4; 2347 parents [98.8%]). By contrast, parents found it difficult to answer whether their child was exceptionally or seriously ill, with 1612 parents (67.9%; Q2) to 1861 parents (78.4%; Q15) answering these questions. Specific questions about the child or adolescent, such as tiredness while eating (Q14; 1940 parents [81.7%]), attention paid to parents (Q16; 2008 parents [84.5%]), reaction to the environment (Q33; 2078 parents [87.5%]), and crying patterns (Q12; 2106 parents [88.7%]) had lower response rates.

**Table 2.** Response Rates, Sensitivity, and Specificity of Identifying Severe Acute Illness in Children Younger Than 2 Years With Fever in Pediatric ED

Question/item	Patients in pediatric ED (N = 354)		
	Response rate, No. (%)	Sensitivity (95% CI), %	Specificity (95% CI), %
Parental worry (Q1)	353 (99.7)	93.7 (87.4-97.4)	9.5 (6.1-13.9)
Exceptionally ill (Q2)	274 (77.4)	59.5 (48.5-70.1)	59.5 (48.3-70.1)
Need for urgent treatment (Q5)	336 (94.9)	74.5 (64.9-82.6)	45.6 (39.2-52.2)
Serious illness impression (Q15)	240 (67.8)	47.0 (35.9-58.3)	65.6 (57.6-73.0)
AIOS ≥11 (<2 y with fever)	343 (96.9)	45.1 (34.1-56.5)	46.9 (39.7-54.2)
Tired (Q3)	328 (92.7)	68.5 (59.0-77.0)	35.5 (29.5-41.9)
Not able to play (Q4)	352 (99.4)	66.4 (56.7-75.1)	36.4 (30.3-40.8)
Feeding difficulty (Q10)	348 (98.3)	55.9 (46.1-65.3)	49.0 (42.4-55.5)
Abnormal crying (Q12)	351 (99.1)	76.4 (67.3-83.9)	32.8 (26.9-39.1)
Drowsy (Q13)	283 (79.9)	70.9 (61.5-79.2)	24.5 (19.2-30.4)
Exhausted with feeding (Q14)	294 (83.0)	27.3 (19.2-36.6)	71.7 (65.5-77.3)
Reduced interaction (Q16)	332 (93.8)	27.3 (19.2-36.6)	79.2 (73.5-84.1)
Abnormal breathing (Q27)	318 (89.8)	58.6 (48.8-67.8)	58.7 (52.2-65.0)
Lethargic (Q32)	311 (87.9)	28.4 (20.2-37.9)	72.5 (66.4-78.1)
Reduced reactivity (Q33)	336 (94.9)	47.3 (37.7-57.0)	62.8 (56.4-68.9)
Status category (Q35)	350 (98.9)	28.4 (20.2-37.9)	66.7 (60.3-72.6)
Crying during questionnaire (Q36)	272 (76.8)	47.3 (37.7-57.0)	64.2 (57.8-70.2)

Abbreviations: AIOS, Acute Illness Observation Scale; ED, emergency department; Q, question.

### Sensitivity of Gut Feeling Questions

Among single questions, moderate to high parental worry (Q1) had the highest sensitivity for identifying severe illness for all children and adolescents (91.0%; 95% CI, 88.3%-93.2%) and the youngest ones (93.7%; 95% CI, 87.4%-97.4%) (Table 2, Figure 2). Parent opinions on the need for treatment for their child (Q5) had a sensitivity of 74.2% (95% CI, 70.3%-77.8%). The sensitivity was low for questions asking whether parents felt something was seriously wrong (Q15) and whether the child or adolescent was exceptionally ill (Q2), at 49.4% (95% CI, 44.3%-54.5%) and 55.7% (95% CI, 50.9%-60.5%), respectively.

### Sensitivity of Specific Pediatric Questions

Specific pediatric questions had low sensitivity in recognizing severe illnesses in children and adolescents. Sensitivities of such questions ranged from 17.8% (95% CI, 14.7%-21.2%) for Q16 to 57.8% (95% CI, 53.4%-61.9%) for Q3 (Figure 2). Among specific pediatric questions, the highest sensitivity was obtained for Q3 (“Is the child clearly tired?”; 57.8%; 95% CI, 53.4%-61.9%) and Q4 (“Is your child well enough to play and socialize as normal?”; 57.6%; 95% CI, 53.4%-61.7%). Specific pediatric questions performed better in young children with febrile illness, with sensitivity ranging from 27.3% (95% CI, 19.2%-36.6%) for Q14 to 76.4% (95% CI, 67.3%-83.9%) for Q12 (Table 2).

### Specificity of Gut Feeling Questions

Among gut feeling questions, Q15 (“Do you feel your child is particularly ill or something is seriously wrong?”) had the best specificity for severe illness, at 72.7% (95% CI, 70.1%-75.2%) (Figure 2) and Q2 (“Is the child exceptionally ill?”) had 67.7% (95% CI, 65.2%-70.1%) specificity. Parental worry (Q1), despite its high sensitivity, had the lowest specificity (17.5%; 95% CI, 15.8%-19.4%).

### Specificity of Individual Pediatric Questions

Of specific pediatric questions, Q16 (“Does your child pay less attention to you than usual when you talk to him/her?”) had the highest specificity (89.1%; 95% CI, 87.6%-90.5%) (Figure 2). The specificity was approximately 85% for Q14 (“Does your child become tired or exhausted while eating?”; 85.4%; 95% CI, 83.7%-87.1%), Q32 (“Is your child more lethargic than usual?”; 85.0%; 95% CI, 83.3%-86.7%), and Q36 (“Has your child cried while you filled in this questionnaire?”; 85.4%; 95% CI, 83.6%-87.0%). Q35, in which parents assessed their child’s health status, had the lowest specificity (43.6%; 95% CI, 41.2%-46.0%).

### Machine Learning and Logistic Regression for Predicting Severe Illness

**Figure 3.**
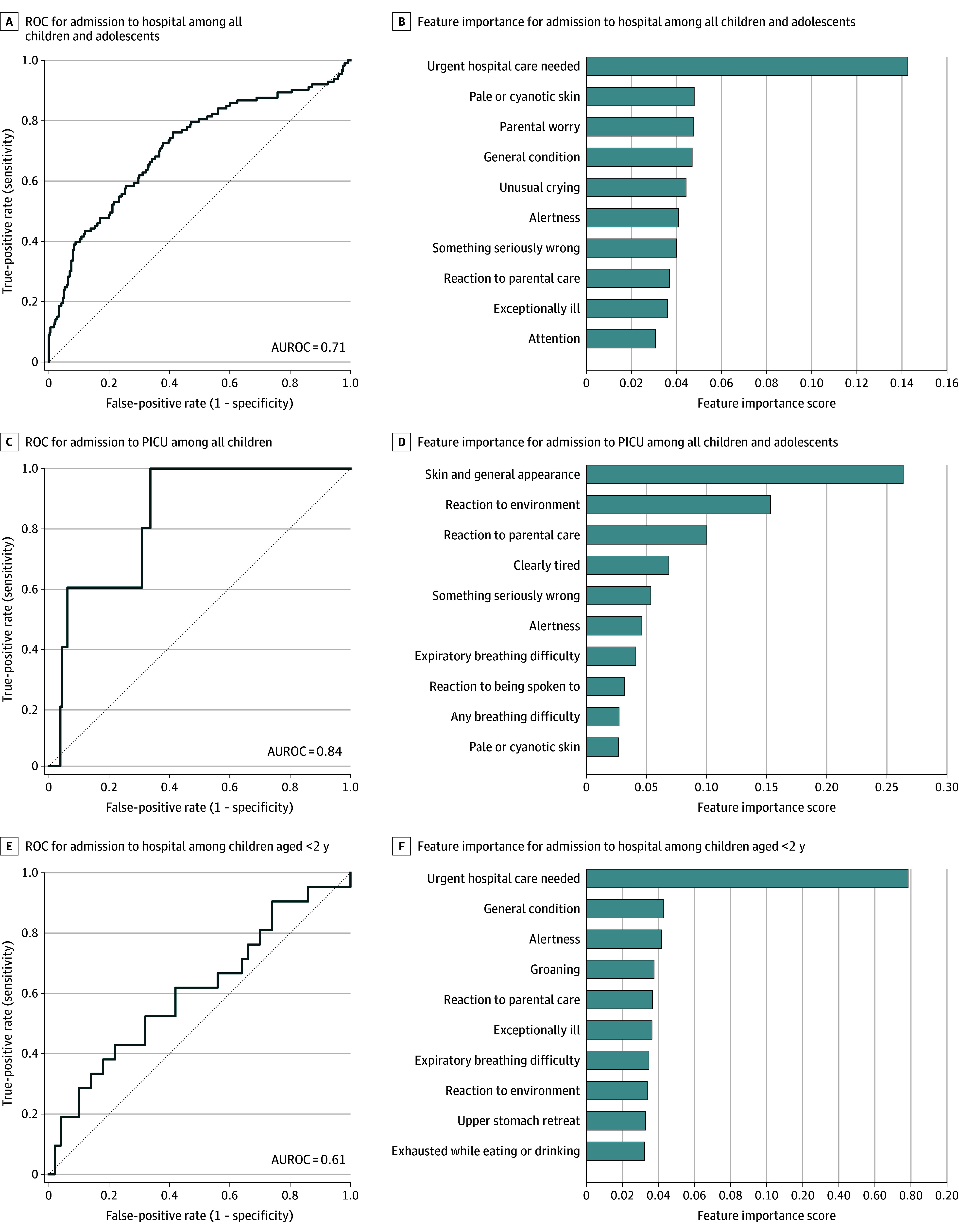
Receiver Operating Characteristic (ROC) Curves and Bar Plots of Feature Importance for Parental Assessment Based on Questionnaire Data Using Gradient-Boosting Algorithm ROC curves were drawn for each estimation, with true-positive rate or sensitivity on the y-axis and false-positive rate or 1 − specificity on the x-axis. Furthermore, the 10 most important questions from the questionnaire for each estimation were depicted as bar plots, with the importance score on the x-axis. AUROC indicates area under the receiver operating characteristic curve; PICU, pediatric intensive care unit.

In a machine learning analysis using all parental questions, hospital admission was predicted with moderate accuracy (AUROC, 0.71; 95% CI, 0.65-0.77). The most important questions for model prediction were Q5 (parental opinion about the need for treatment; feature importance score, 0.141), alongside Q1 (parental worry; feature importance score, 0.047) and Q35 (general condition; feature importance score, 0.046) (Figure 3A-B). Admission to the ICU was predicted with similar accuracy (AUROC, 0.84; 95% CI, 0.71-0.98), with Q31 (skin and general appearance) being the most important question, alongside Q33 (reaction to environment) and Q18 (reaction to treatment) (Figure 3C-D). In children younger than 2 years with febrile illness, machine learning performed poorly at predicting hospital admission (AUROC, 0.61; 95% CI, 0.46-0.76) (Figure 3E-F).

In the logistic regression analysis (eTable 3 in Supplement 1) using all parental questions, the following parental questions were associated with a hospital admission: the child or adolescent appeared exceptionally ill (Q2), required urgent treatment (Q5), or paid less attention to the parent (Q16) or the parent felt that something was seriously wrong (Q15). Parental questions about the child or adolescent’s ability to play and socialize (Q4) or appearing exceptionally ill (Q2) and parental worry (Q15) were associated with admission to the PICU.

### Exploratory Parental Score in Recognizing Severe Illness

When combining the 3 most important questions from the machine learning model (Q1, Q5, and Q35), exploratory parental score showed poor diagnostic test performance in identifying children and adolescents needing hospital admission, with an AUROC of 0.64 (95% CI, 0.61-0.66) (eTable 4 in Supplement 1). The sensitivity varied from 40.3% (95% CI, 36.2%-44.4%) to 66.7% (95% CI, 46.0%-83.5%) and the specificity from 72.3% (95% CI, 70.5%-74.1%)to 79.4% (95% CI, 77.5%-81.2%) with the optimal cutoff value (score ≥10) for illness requiring hospital admission. For PICU admission, the AUROC was 0.73 (95% CI, 0.68-0.78); the sensitivity for the best cutoff value of 10 ranged from 66.7% (95% CI, 46.0%-83.5%) to 77.8% (95% CI, 57.7%-91.4%) and the specificity from 62.7% (95% CI, 60.7%-64.7%) to 75.2% (95% CI, 73.4%-76.9%).

## Discussion

In this diagnostic study involving 2375 child or adolescent–parent pairs in a pediatric ED, parental ability to recognize their child’s severe illness was evaluated using a simple questionnaire completed upon arrival. Questions assessing the parental gut feeling demonstrated high sensitivity for identifying severe acute illness in their children. Accordingly, the most important parental observations for predicting severe illness in our machine learning analysis were those concerning the child or adolescent’s general condition, the perceived need for urgent medical care, and parental worry. The specificity of the parental questions with high sensitivity, however, was low. This was associated with poor overall diagnostic performance when a set of questions was combined in the machine learning model or exploratory parental score.

Identifying severe acute illness is difficult in young children.^22^ Severe acute illnesses in children and adolescents often start with a nonspecific presentation before progressing to a life-threatening condition.^23^ Most studies analyzing parental assessment of children with acutely illness have used a qualitative design or included only children with febrile illness.^9,10,24,25^ Findings of this study align with previous primary care studies showing that parental gut feeling is a red flag for a child or adolescent’s severe illness.^8,9^ More importantly, our study shows that while parental worry or gut feeling questions had excellent sensitivity in recognizing a severe illness, the specificity of most parental questions was low. This was associated with poor diagnostic performance measured by AUROC in a machine learning model or in an exploratory parental score. These findings suggest that while parental concern identifies children and adolescents who may need urgent evaluation, it also generates many false positives and therefore cannot be used as a stand-alone decision tool in busy ED settings.

Illness severity scoring systems have been developed to predict severe illnesses in children with acute illness.^4,5,6,26^ Some efforts have also been made to create a scoring system for parental use.^11,27^ Parents have been reported to recognize their child’s severe illness even before nurses and doctors.^28^ Our study shows that this valuable parental ability may be lost if families are exposed to a well-intended but too-complicated set of questions. Thus, the approach emphasizing simple parental questions may enhance the recognition of children and adolescents with severe illness.

The digitization of health care has the potential to transform the management of severe childhood illnesses. In a previous pilot validation study^29^ involving 294 children with influenza-like symptoms, parents used a web-based decision-support tool to determine whether their child required immediate care in an ED. The algorithm’s sensitivity was high, at 93%, but its specificity was poor, at 13%. In another study,^30^ 126 parents used the smartphone application Should I See a Doctor in a primary care setting; the application had a sensitivity and specificity of 84% and 74%, respectively, compared with the triage nurse’s advice. Machine learning is currently considered for many aspects of pediatric care, including clinical decision-making.^31^ Machine learning is an effective tool if the dataset is valid and reliable. Our results indicate that parental triage questions regarding children and adolescents with acute illness must be carefully evaluated before use in clinical decision-making. Notably, parental assessment of young children appeared challenging in our study. This suggests that algorithms intended for children younger than 2 years require targeted validation before implementation.

### Strengths and Limitations

This study has several strengths. We recruited a large cohort of children and adolescents upon arrival at a pediatric ED during 2 epidemiological years. This cohort represented different age groups and had mild and severe diseases, reflecting ED triage in clinical practice. We reviewed medical records of all patients to obtain accurate medical histories and determine their health outcomes. We used multivariable analysis and machine learning to evaluate whether certain combinations of questions were associated with better predictability.

This study also has limitations. Even though we reached the predefined sample size, some parents did not participate in the study, which could affect the generalizability of the results. We did not collect data on parent educational level or socioeconomic status. However, a previous study,^32^ showed that parent age and level of education were not associated with their ability to predict severe bacterial infection in their children. Poor parent-child interaction, not measured in our study, has been shown to associate with low specificity of clinical judgment and overuse of resources.^33,34^ In addition, because only Finnish-speaking families were eligible, our findings may not fully generalize to linguistically diverse populations. Additionally, although the reference standard (eg, hospital admission, PICU admission, and the need for intravenous therapy) was determined independently of the questionnaire, it is possible that brief triage interactions, such as comments or observations made by the triage nurse, may have influenced how some parents interpreted their child’s condition when completing the questionnaire. Such subtle influence is unlikely to have systematically affected clinical decisions but may have minimally influenced parental responses, potentially inflating diagnostic accuracy estimates.

## Conclusions

In this diagnostic study of children and adolescents with acute illness presenting to the ED, parental worry was a highly sensitive indicator of severe illness but had low specificity. While parental concern is an important red flag, it should be interpreted alongside clinical assessment to avoid unnecessary escalation of care. Digital tools designed for parental use require careful validation before implementation.

## Notes

Supplement 1. **eAppendix 1.** Questionnaire for parents of children and adolescents with acute illness **eAppendix 2.** Acute Illness Observation Scale (AIOS) **eTable 1.** Hyperparameter tuning for each algorithm during model testing **eTable 2.** Best-performing models for each analysis **eTable 3.** Adjusted odds ratios for a severe illness requiring treatment in the hospital ward or PICU based on parental triage questionnaire **eTable 4.** Construction of 3-item parental score

Supplement 2.
Data Sharing Statement
