# Supplementary material for: Parental Ability to Identify Severe Illnesses in Their Children
Source: JAMA Netw Open. 2026 Feb 17;9(2):e2559998. doi: 10.1001/jamanetworkopen.2025.59998 (PMC12914493; doi:10.1001/jamanetworkopen.2025.59998)
Supplement: Supplement 1. — eAppendix 1. Questionnaire for parents of children and adolescents with acute illness eAppendix 2. Acute Illness Observation Scale (AIOS) eTable 1. Hyperparameter tuning for each algorithm during model testing eTable 2. Best-performing models for each analysis eTable 3. Adjusted odds ratios for a severe illness requiring treatment in the hospital ward or PICU based on parental triage questionnaire eTable 4. Construction of 3-item parental score [file jamanetwopen-e2559998-s001.pdf]

Supplemental Online Content

Pöyry H, Turunen J, Ritola E, et al. Parental ability to identify severe illnesses in their children. *JAMA Netw Open*. 2026;9(2):e2559998. doi:10.1001/jamanetworkopen.2025.59998

eAppendix 1. Questionnaire for parents of children and adolescents with acute illness

eAppendix 2. Acute Illness Observation Scale (AIOS)

eTable 1. Hyperparameter tuning for each algorithm during model testing

eTable 2. Best-performing models for each analysis

eTable 3. Adjusted odds ratios for a severe illness requiring treatment in the hospital ward or PICU based on parental triage questionnaire

eTable 4. Construction of 3-item parental score

This supplemental material has been provided by the authors to give readers additional information about their work.

© 2026 Pöyry H et al. *JAMA Network Open*.

eAppendix 1: Questionnaire for parents of acutely ill children

Form completed on:
______________ date __________________ time

Birth date and social security number:____
The child's first and last name:____

When did the symptoms of this disease first appear in your child?

______________ date __________________ time (your own best estimate)
Your estimate of travel time to this emergency department (distance between the child's home or other place of location and the hospital):
________ h ________ min ________ km
Describe very briefly what you consider to be the child's most important symptom or your biggest concern at the moment? Write your answer below in your own words.
____
____

1. **As a parent, how worried are you about your child's health at the moment? *Put an X* in the box next to the alternative closest to your opinion.**

Not at all worried	□
Slightly worried	□
Moderately worried	□
Very worried	□
Exceptionally worried	□
I don't know	□

2. **In your opinion, is the child exceptionally ill?**

Yes	□
No	□
I don't know	□

3. **In your opinion, is the child clearly tired?**

Yes	□
No	□
I don't know	□

4. **Is your child well enough to play and socialize as normal?**

Yes	□
No	□
I don't know	□

5. **As a parent, what do you think about your child's need of treatment? *Put an X* in the box next to the alternative that is closest to your own opinion.**

Does not necessarily need to be assessed by a doctor right away	□
Needs to be assessed by a doctor now, but could then go back home	□
Needs to be assessed by a doctor now and needs medication and treatment, but could then go back home	□
Needs hospital treatment in the ward now	□
Needs particularly careful monitoring and effective hospital treatment now	□
I don't know	□

© 2026 Pöyry H et al. *JAMA Network Open*.

6. **In your opinion, was it easy to get help for your child during this illness? Choose the alternative that is closest to your own opinion.**

Very easy	◻
Easy	◻
Not easy, but not difficult	◻
Relatively difficult	◻
Very difficult, and it felt that it took a long time to get treatment, or it did not go well	◻
I cannot say	◻

7. **During this illness, before coming to the emergency department, I have been in contact with or tried to be in contact with the following:** ***Put an X*** **next to the most applicable alternative.**

	**No**	**Did not succeed**	**Once**	**Twice**	**3 x**
Phone counseling	◻	◻	◻	◻	◻
Nurse clinic	◻	◻	◻	◻	◻
Health center doctor	◻	◻	◻	◻	◻
GP in private sector	◻	◻	◻	◻	◻
Family Pediatrician	◻	◻	◻	◻	◻
Other doctor	◻	◻	◻	◻	◻
Ambulance staff	◻	◻	◻	◻	◻
Internet search ◻	◻	◻	◻	◻	◻

8. **Has your child been assessed by a doctor for this illness in the previous 48 hours?**

Yes	◻
No	◻

9. **When did you first get the feeling that this illness might require a doctor?**

______________ date __________________ time (your own best estimate)

10. **Has your child had problems breastfeeding or drinking, or has the amount the child has drunk been smaller than usual?**

Yes	◻
No	◻
I don't know	◻

if you answered ***yes***: **How much breastmilk or other liquids has the child taken? Choose the most applicable alternative:**

Only slightly less than usual (more than two thirds of the usual amount)	◻
About half of the usual amount (one third–two thirds of the usual amount)	◻
Very little (less than one third of the usual amount)	◻

11. **Has the child passed less urine than normal?**

Yes	◻
No	◻
I don't know	◻

© 2026 Pöyry H et al. *JAMA Network Open*.

12. Has your child cried in a manner that is unusual to him/her (exceptionally shrill, complaining or similar)?
Put an X **next to the alternative that is closest to your own opinion.**

Yes ◻
No ◻
I don't know ◻

13. Has your child been unusually drowsy (less alert than usual) while awake?

Yes ◻ No ◻
I don't know ◻

if you answered <u>yes</u>: **choose the most applicable alternative among the following**

Drowsy at times (alert most of the time) ◻
Drowsy most of the time (alert at times) ◻
Drowsy all the time (not alert at all) ◻

14. Does your child become tired or exhausted while eating or drinking?

Yes ◻
No ◻
I don't know ◻

15. Do you feel that your child is particularly ill or that there is something seriously wrong?

Yes ◻
No ◻
I don't know ◻

16. Does your child pay less attention to you than usual when you talk to him/her?

Yes ◻
No ◻
I cannot say ◻

17. How has your child reacted when being spoken to?

Smiles or reacts in other ways that are characteristic of him/her ◻

© 2026 Pöyry H et al. *JAMA Network Open*.

Brief smile or alerts briefly □
Does not smile, or the face is anxious, dull or expressionless □
No alerting □
I cannot say □

18. How has your child reacted when being cared for by you? Choose the alternative that is closest to your own opinion.

Is content, does not cry □ Cries
briefly and then calms down □
Cries off and on □ Cries
all the time □ Hardly responds
□
I cannot say □

19. Assess the quality of your child's crying by choosing the alternative that best describes it:

The child is content and does not cry □
The crying is strong with normal tone □
The crying is whimpering or sobbing □
The crying is weak or moaning □
The crying is high-pitched □
I cannot say □

20. Has your child had a fever during this illness?

Yes □
No □
Maybe □
I don't know □

21. What is the highest body temperature measured during this illness? _____________

I don't know □
Not measured, but the child felt feverish □
Not measured, no fever □

22. Has your child had convulsions or similar seizures during this illness?

Yes □ No □
I don't know □

23. Has your child had diarrhea during this illness?

Yes □ No □
I don't know □

if you answered yes: **please answer these additional questions as well**
Duration of diarrhea up to now _____ days_____ hours
How many times per day (24 h) has the child had diarrhea stools?__________
Has there been blood in the stools? Yes □ No □ I don't know □

© 2026 Pöyry H et al. *JAMA Network Open*.

24. Has your child vomited during this illness (exclude normal bringing up of milk after feeding)?
Yes □
No □
I don't know □

if you answered yes: **choose the most applicable alternative among the following**
Duration of vomiting up to now _____ days_____ hours
How many times has the child vomited per day (24h) ___________
Does the child vomit at least half of the amount eaten? Yes □ No □
Is the vomit green in color? Yes □ No □
Did the vomiting begin after surgery? Yes □ No □

25. Before coming to the emergency department, have you noticed that your child could have a groin hernia (a bulge in the groin area)?
Yes □ No □
I don't know □

26. Does your child have a cough? Yes
□ No □
I don't know □

if you answered yes:
Does the cough come in fits? Yes □ No □ I don't know □

27. In your opinion, is your child's breathing abnormal, labored or difficult?
Yes □
Possibly yes □
No □
I don't know □

28. Has your child been given any medication to help him/her breathe at home or in some other place of care before coming to the emergency department?
Yes □
No □
I don't know □

29. What is the child's skin color?
Normal □
Normal at the moment, but has looked very pale during the previous 24 hours □
Pale or bluish feet or hands □
Very pale skin □
Cyanotic or mottled or ashen □ Grey or
white and mottled □
I cannot say □

30. Have you noticed a new skin rash on your child's skin, covering an area of at least 5 x 5 cm, that you have not seen before?

© 2026 Pöyry H et al. *JAMA Network Open*.

Yes ☐
No ☐
I don't know ☐

31. What do you think about your child's skin and general appearance?
Normal: the skin is normal, the eyes are normal (not sunken in), and the mucous membranes in the mouth are moist ☐
The skin and eyes are normal, but the mucous membranes are slightly dry ☐
The eyes are sunken in ☐
The eyes are sunken in, the skin is doughy or tented, and the mouth is dry ☐
I cannot say ☐

32. Is your child more lethargic than usual?
Yes ☐ No ☐
I don't know ☐

33. In your opinion, does your child react less to what is happening around him/her than usual?
Yes ☐ No ☐
I cannot say ☐

34. Assess your child's alertness by choosing the most applicable alternative that is closest to your own opinion among the following:
Awake and alert ☐
Sleeps but is easy to wake up ☐
Keeps falling asleep at times but wakes up by himself/herself ☐
Sleeps and takes a longer time than usual to wake up ☐
Sleeps all the time, rarely wakes up, or is hard to wake up ☐
I cannot say ☐

35. Finally, assess your child's status by choosing the most applicable alternative among the following:
Plays or acts otherwise as usual ☐
Sleeps or is drowsy ☐
Restless or irritable ☐
Lethargic, confused, or clearly less responsive than usual ☐
I cannot say ☐

36. Has your child cried while you filled in this questionnaire?

Yes ☐
No ☐
I cannot say ☐

© 2026 Pöyry H et al. *JAMA Network Open*.

eAppendix 2. Acute Illness Observation Scale (AIOS)

Observation scale adapted from McCarthy PL, et al. Pediatrics. 1982;70(5):802-809.

Observation Item	Normal (1)	Moderate Impairment (3)	Severe Impairment (5)
**Quality of cry**	Strong with normal OR tone OR content and not crying	Whimpering sobbing	Weak OR moaning OR high-pitched
**Reaction to parent stimulation**	Cries briefly then stops OR content and not crying	Cries off and on	Continual cry OR hardly responds
**State variation**	Awake stays awake / if asleep, wakes quickly	Eyes close briefly, then awake OR awakes with prolonged stimulation	Falls to sleep OR will not rouse
**Color**	Pink	Pale extremities OR OR acrocyanosis	Pale OR cyanotic mottled OR ashen

© 2026 Pöyry H et al. *JAMA Network Open*.

eTable 1. Hyperparameter tuning for each algorithm during model testing.

Algorithm	Parameter	Values
Random Forest	N. of estimators<br>Criterion<br>Max depth<br>Min. samples split<br>Min. samples leaf<br>Min. weight fraction leaf<br>Max. features<br>Bootstrap	100-2000<br>Gini, entropy, log-loss<br>5-10<br>2-20<br>1-20<br>0.0, 0.1, 0.2, 0.3, 0.4, 0.5<br>Sqrt, log2, none<br>True, False
Linear SVC	Loss<br>Tol<br>C<br>Max iter	Hinge, squared hinge<br>0.001, 0.01, 0.1<br>1-20<br>100-2000
Gradient Boosting	Loss<br>Learning rate<br>N. of estimators<br>Criterion<br>Max depth<br>Min samples split<br>Min. samples leaf<br>Max features	Log-loss, exponential<br>0.01, 0.1, 0.2<br>100-2000<br>Friedman mse, squared error<br>5-10<br>2-20<br>1-20<br>Sqrt, log2, none
KNeighbors	N. of neighbors<br>Weights<br>Algorithm<br>Leaf size<br>Metric	5-100<br>Uniform, distance, none<br>Ball tree, kd tree<br>5-100<br>Euclidean, Manhattan, Minkowski
Extra Trees	N. of estimators<br>Criterion<br>Max depth<br>Min. samples split Min. samples leaf<br>Min. weight fraction leaf<br>Max. features<br>Bootstrap	100-2000<br>Gini, entropy, log-loss<br>5-10<br>2-20<br>1-20<br>0.0, 0.1, 0.2, 0.3, 0.4, 0.5<br>Sqrt, log2, none<br>True, False

© 2026 Pöyry H et al. *JAMA Network Open*.

eTable 2. The best-performing models for each analysis.

Analysis	Used algorithm	Used parameters	Used parameters, values
All children, admission to hospital	Gradient Boosting	Loss<br>Learning rate<br>N. of estimators<br>Criterion<br>Max depth<br>Min samples split<br>Min. samples leaf<br>Max features	Exponential<br>0.01<br>724<br>Squared error<br>5<br>6<br>16<br>Log2
All children, intensive care	Gradient Boosting	Loss<br>Learning rate<br>N. of estimators<br>Criterion<br>Max depth<br>Min samples split<br>Min. samples leaf<br>Max features	Exponential<br>2<br>505<br>Squared error<br>6<br>19<br>7<br>Log2
Children <2 years, admission to hospital	Gradient Boosting	Loss<br>Learning rate<br>N. of estimators<br>Criterion<br>Max depth<br>Min samples split<br>Min. samples leaf<br>Max features	Exponential<br>0.01<br>279<br>Squared error<br>7<br>17<br>1<br>Sqrt

© 2026 Pöyry H et al. *JAMA Network Open*.

eTable 3. Adjusted Odds Ratios for a severe illness requiring treatment in the hospital ward (A) or PICU (B) Based on Parental Triage Questionnaire *aOR = adjusted Odds Ratio; CI = Confidence Interval; PICU = Pediatric Intensive Care Unit

A. A severe illness requiring treatment in the hospital

Question	aOR	95 % CI
Q2 Exceptionally ill	1.88	1.22-2.91
Q3 Clearly tired	0.65	0.45–0.93
Q5 Need for urgent treatment	1.93	1.38–2.71
Q15 Feeling something is seriously wrong	1.94	1.26–2.97
Q16 Child pays less attention to the parent	1.90	1.25–2.87

B. A severe illness requiring treatment in the PICU

Question	aOR	95 % CI
Q2 Exceptionally ill	12.80	1.45–112.75
Q3 Clearly tired	0.13	0.03–0.52
Q4 Not able to Play/Socialize	9.81	1.04–92.92
Q16 Child pays less attention to the parent	7.19	1.65–31.23

© 2026 Pöyry H et al. *JAMA Network Open*.

eTable 4. Construction of the three-item parental score

Question	Response option	Score
Q1 How worried are you about your child's state of health now?	Not at all worried<br>Slightly worried<br>Moderately worried<br>Very worried<br>Exceptionally worried<br>I don't know	1<br>2<br>3<br>4<br>5<br>Excluded / 1 / 5
Q5 What do you think about your child's need of treatment?	Does not necessarily need to be assessed.<br>Needs assessment now but could go home Needs assessment + medication but home afterwards<br>Needs hospital treatment in the ward<br>Needs careful monitoring + effective treatment<br>I don't know	1<br>2<br>3<br>4<br>5<br>Excluded / 1 / 5
Q35 assess your child's status	Plays or acts as usual<br>Sleeps / drowsy<br>Restless / irritable<br>Lethargic / confused / less responsive<br>I don't know	1<br>2<br>3<br>4<br>Excluded / 1 / 5

© 2026 Pöyry H et al. *JAMA Network Open*.
