# Supplementary material for: Parental Ability to Identify Severe Illnesses in Their Children
Source: JAMA Netw Open. 2026 Feb 17;9(2):e2559998. doi: 10.1001/jamanetworkopen.2025.59998 (PMC12914493; doi:10.1001/jamanetworkopen.2025.59998)
Supplement: Supplement 2. — Data Sharing Statement [file jamanetwopen-e2559998-s002.pdf]

Data Sharing Statement

Pöyry. Parental Ability to Identify Severe Illnesses in Their Children. *JAMA Netw Open*. Published online February 17 2026.
doi:10.1001/jamanetworkopen.2025.59998

Data
Data available: Yes
Data types: Deidentified participant data
How to access data: Upon reasonable request to the corresponding author, hilla.poyry@pohde.fi The machine learning data will be submitted upon the publication of the manuscript. During the review process, it can be accessed from the following link: https://zenodo.org/records/15512119?preview=1&token=eyJhbGciOiJIUzUxMiJ9.eyJpZCI6IjcwZGZhM2ZmLTI1MTAtNDNlOC1iY2E1LTJkNTNjMDM2OWQ4NSIsImRhdGEiOnt9LCJyYW5kb20ifIHthWXG0bGyg4Yg
When available: With publication

Supporting Documents
Document types: None

Additional Information
Who can access the data: Deidentified individual participant data (with data dictionary) will be made available upon reasonable request to qualified researchers for purposes of verifying study findings or conducting patient safety–related analyses. Requests will be evaluated by the corresponding author and study team. For the machine learning data, it will be submitted upon the publication of the manuscript in Zenodo.
Types of analyses: Deidentified individual participant data (with data dictionary) will be made available upon reasonable request to qualified researchers for purposes of verifying study findings or conducting patient safety–related analyses. Requests will be evaluated by the corresponding author and study team
Mechanisms of data availability: A data access agreement will be required to ensure appropriate use
Any additional restrictions: The machine learning data will be submitted upon the publication of the manuscript. During the review process, it can be accessed from the following link: https://zenodo.org/records/15512119?preview=1&token=eyJhbGciOiJIUzUxMiJ9.eyJpZCI6IjcwZGZhM2ZmLTI1MTAtNDNlOC1iY2E1LTJkNTNjMDM2OWQ4NSIsImRhdGEiOnt9LCJyYW5kb20ifIHthWXG0bGyg4Yg
